# Social network diagnostics: a tool for monitoring group interventions

**DOI:** 10.1186/1748-5908-8-116

**Published:** 2013-10-01

**Authors:** Sabina B Gesell, Shari L Barkin, Thomas W Valente

**Affiliations:** 1 Department of Social Sciences and Health Policy, Division of Public Health Sciences, Wake Forest School of Medicine, Medical Center Boulevard, Winston-Salem, NC 27157, USA; 2 Maya Angelou Center for Health Equity, Wake Forest School of Medicine, Medical Center Boulevard, Winston-Salem, NC 27157, USA; 3 Division of General Pediatrics, Department of Pediatrics, Vanderbilt School of Medicine Nashville, Nashville, TN, USA; 4 Institute for Prevention Research, Department of Preventive Medicine, Keck School of Medicine, University of Southern California, Los Angeles, CA, USA

**Keywords:** Social network analysis, Behavior change, Group interventions, Obesity

## Abstract

**Background:**

Many behavioral interventions designed to improve health outcomes are delivered in group settings. To date, however, group interventions have not been evaluated to determine if the groups generate interaction among members and how changes in group interaction may affect program outcomes at the individual or group level.

**Methods:**

This article presents a model and practical tool for monitoring how social ties and social structure are changing within the group during program implementation. The approach is based on social network analysis and has two phases: collecting network measurements at strategic intervention points to determine if group dynamics are evolving in ways anticipated by the intervention, and providing the results back to the group leader to guide implementation next steps. This process aims to initially increase network connectivity and ultimately accelerate the diffusion of desirable behaviors through the new network. This article presents the Social Network Diagnostic Tool and, as proof of concept, pilot data collected during the formative phase of a childhood obesity intervention.

**Results:**

The number of reported advice partners and discussion partners increased during program implementation. Density, the number of ties among people in the network expressed as a percentage of all possible ties, increased from 0.082 to 0.182 (p < 0.05) in the advice network, and from 0.027 to 0.055 (p > 0.05) in the discussion network.

**Conclusions:**

The observed two-fold increase in network density represents a significant shift in advice partners over the intervention period. Using the Social Network Tool to empirically guide program activities of an obesity intervention was feasible.

## Background

Many behavioral interventions designed to improve health outcomes are delivered in group settings. For example, Alcoholics Anonymous participants meet in groups to discuss their addiction issues and get support from other group members to abstain from alcohol use. Many other group interventions have been created and tested with the intent of fostering support and interaction among group members to help initiate and sustain positive behavior changes. To date, however, few evaluations of group-level interventions have been conducted that explicitly monitor whether group interaction evolves in a way that supports program outcomes [1,2].

The purpose of this article is to present a model and new diagnostic tool for group interventions that assesses group interactivity and empirically directs changes during program implementation to increase group cohesion, which should theoretically, accelerate behavior change. The approach is based on social network analysis (SNA) and entails making network measurements of the groups at strategic points of the intervention to determine if group dynamics are evolving in ways specified or anticipated by the intervention. The network diagnostic tool generates measurements that are converted to a set of individual and group metrics that indicate how social ties and social structure are changing within the group and can be used to guide program activities. This article presents the Social Network Diagnostic Tool and, as proof of concept, pilot data collected during the formative phase of a childhood obesity intervention.

### Social network analysis

SNA is a set of theories and techniques used to understand how social relationships (*e.g.*, friendship, advice seeking, reputation) influence behaviors [3-5]. SNA allows us to see a whole group of individuals and their interconnectedness. The influence of social networks for many behaviors has been studied, including contraception [6], risk for HIV/AIDS HIV/STDs [7], smoking [8,9], physician behavior [10,11], obesity [12], physical activity [13], and substance use [1,14,15]. The evidence indicates that existing social networks are important influences on health-related behaviors (*i.e.*, children adjust their physical activity level to emulate their friends) and that social networks are an outcome of health related behaviors in the form of network selection (*i.e.*, smokers choose smoking friends) [16,17].

Rooted in graph theory and linear algebra, SNA provides a set of tools for quantifying a person’s position within a social hierarchy and is useful for characterizing people’s social influences, such as the number or percent of their close friends who engage in certain behaviors [18-20]. Social networks influence behavior through several theoretical mechanisms. Networks provide information for behavior change, they can influence perception of social norms, they provide how-to knowledge, and they can provide social comparisons.

Social networks differ from social support. Methodologically, social support is measured from the respondent’s perspective to assess the support (*e.g.*, emotional, cognitive, tangible support) an individual perceives to have from others. Social networks, in contrast, typically measure the presence or absence of mutual friendships and other task- or work-oriented relationships (which may or may not provide support) and treats the bi-directional ties themselves as objects of study [21]. In this paper, we focus only on social networks.

When examining social networks, network metrics are calculated at both the individual and network level [3]. At the individual level, SNA can be used to determine each person’s position in the group based on the relationships reported by the other group members, such as whether or not a person is connected to others, how central that person is in the larger network, and whether or not a person belongs to a subgroup. Thus, individual measures such as the number of ties or the number of ties to others with a similar attribute (*e.g.*, same sex) indicate properties of the individual and may be correlated with individual outcomes. At the group level, SNA can be used to measure the emergence of connectedness among people, the overall structure of the network, and how these relationships influence health behaviors. Thus, network level metrics, such as the density (the number of ties among people in the network expressed as a percentage of all possible ties) or transitivity of the network (the tendency for a friend of a friend to become a friend), indicate properties of the whole network and may be correlated with group outcomes.

In this study we calculate several individual and network level metrics, which are widely used to describe networks [3,5], and show how the information contained in this analysis can be used by intervention staff during program implementation to better integrate members into the group. Table 1 summarizes the diagnostics we propose to be most informative for program monitoring, along with suggested thresholds. We use both individual- and network-level metrics. The individual metrics are: isolates, degree, and reciprocity; and the network metrics are sub-groups, density, centralization, transitivity, and cohesion. There are many other metrics available in SNA software (*e.g.*, UCINET, Pajek, Statnet), and this proposed list is not meant to be exhaustive or exclusive, merely a recommendation of the ones most likely to have immediate effects on individual and group processes as well as those for which intervention recommendations could be made, guided by basic research on network properties.

**Table 1:** Network diagnostics tool

**Metric**	**Threshold**	**Description**	**Rationale**	**Teaching methods thought to improve network structure**
Isolates	Value should be equal to 0	A person not connected to anyone.	We want everyone to be connected to at least one other person in the group. Connections are required for behavior transmission. Interventionist should make sure the isolates do not feel excluded.	1. Interventionist is instructed to pair isolates with highly connected group members in small group activities in session.
				2. Interventionist is instructed to call on isolates first to answer questions in session with the goal of not letting them fade into the background. Interventionist to refer back to what the isolate said to show their input is valued.
				3. Interventionist is instructed to catch the isolate alone (if possible), and to check in with her to let her know we care and ask how GROW is going for her, without putting her on the spot publically.
Degree	Value should be greater than 1	The number of ties coming from each person and going to each person.	As an extension of isolates, we also want to know how well connected each person is, whether they have links coming from them and going to others.	1. Interventionist is instructed to pair highly connected group members with others in small group activities in session.
Reciprocity	Values should be >0.50; (Examine Reciprocity and Reciprocity Non Nulls) ^d^	The extent to which ties are reciprocated.	If reciprocity is low, existing ties are weak. Transmission of behavior is more likely with strong ties.	1. Interventionist is instructed to pair non-reciprocated links: If A sends a tie to B, but B does not send a tie to A, then Interventionist will pair A and B in small group activities in session.
Components/ subgroups	Value should be equal to 0	The presence of disconnected groups in the networks.	The presence of components indicates a splintering of the group which might indicate fractions. Interventionist should consider building bridges between fractions/sub-groups.	1. Interventionist is instructed to pair members from different subgroups in small group activities in session (create bridges).
				2. Interventionist is instructed to make sure small groups do not split along these lines. If they do, interventionist will reassign members.
Density	Value should be >0.15 ^d^ but <0.50 [3]	The extent to which members are connected: number of ties present divided by number of possible ties.	If density is too low, the intervention is not building connections. Connections are required for behavior transmission. Interventionist should consider including activities that create more connectivity in the group.	1. Interventionist is instructed to begin each session with an interactive, personalized, community-building ice breaker.
				2. Interventionist is instructed to uses a beach ball (“talking stick”) to give each participant the opportunity to be part of the conversation.
				3. Interventionist is instructed to helps group establish a stronger group identity: At each session, the facilitator will emphasize (a) the larger GROW family as a support network with a shared mission of improving health; (b) that there needs to be balance in what people put in and get out of the group; and that the more families invest in each other, the more they can get from the group; (c) that all parents need and deserve support in the face of our obesogenic environment, and (d) that this is the most dedicated group of parents committed to the issue of pediatric obesity prevention.
				4. The interventionist is instructed to spend 15 minutes so that each member is offered the opportunity to share some relevant aspect of their lives and expectations (personal information). The interventionist gives instruction on the technique of mutual invitation process to structure such sharing.
				5. Interventionist facilitates making and meeting a shared common goal.
Centralization	Values should be <0.25^d^ (Examine relative values on degree, closeness, betweenness to identify central nodes.)	The extent to which the network is focused on one or a few people.	An overly centralized network indicates that one person occupies a position of critical importance in the network (i.e., has more social power). Interventionist should either work to decentralize the network, giving others important roles, or work with that central person to make sure they support the intervention.	1. Interventionist is instructed not to let the central node start conversations, be the first person to answer a question, lead goal review, or lead group activity. If a central person asks to lead a group activity, the interventionist redirects so that member on the periphery can take on that role.
				2. Interventionist is instructed to avoid pairing central nodes with isolates.
Transitivity ^e^	Values should be >0.3^d^[22]	The extent to which two of a person’s friends are friends with each other.	Transitivity provides a measure of cohesion in a network. We expect relationships to become transitive; if this is too low it might indicate a hierarchical structure in the network. Interventionist should bring triads together.	1. Interventionist is instructed to bring triads together in activities in session. If A is friends with B and C, Interventionist will connect B and C.
Cohesion ^e^ (average of inverse distances)	Values should be <0.50 (±.25) ^e^	The extent to which individuals in the group are directly connected. The path between two people is the length of the path connecting them.	We want higher values which indicate greater group cohesion to facilitate the transmission of behavior. Cohesion is a mediator of group formation and maintenance.	1. Interventionist is instructed to challenge the group to make and meet a shared common goal (weekly wellness challenge: 15 minutes of walking per day). Group will track their minutes during the week and total the group’s minutes to see combined efforts next session. Group will track success each session, increase daily goal in subsequent sessions.

This table describes the network diagnostic metrics and thresholds used, why they were selected, along with the corresponding “menu of teaching methods” that can be provided to group leaders to increase group cohesion.

^d^ Threshold reflects expert recommendation.

^e^ Threshold is a guess and given a wide confidence interval.

### Growing right onto wellness (GROW)

GROW is an ongoing group-level behavioral intervention to prevent childhood obesity. It occurs at public community recreation centers for high-risk parent-preschool child (ages three to five years) dyads. GROW is based on a conceptual model that childhood growth patterns are affected over time at sensitive windows of development by both micro- and macro-level systems [23,24]. The micro-level system includes personal characteristics ranging from genetic profiles to individual attitudes and behaviors; whereas the macro-level system ranges from social networks to public policies. The GROW intervention focuses on the family, recruiting an index parent–child dyad, and connecting that dyad to the larger built environment. This built environment serves as a community-centered location to build healthy lifestyle skills (both routine physical activity and nutritional habits). During the first (intensive) phase of the intervention, families attend skills-building sessions together in small groups for twelve weeks. We think the new social networks that form during these group sessions will operate as a mediating variable on study outcomes.

While many obesity interventions occur in a group setting, underlying group structure and group processes are not documented in the scientific literature. Our own research demonstrated that group intervention sessions increased group connectivity. In a recently published study, we found that a new social network evolved among families participating in another pediatric obesity group-based prevention trial [2]. Moreover, these new social ties formed in a predictable manner: mothers selectively formed new friendships with other mothers based on child body mass index [2]. This reveals the tendency for mothers to form new friendships with mothers whose children have similar body types. We know that both the formation of new friendships, and the selective formation of friendships have the potential to facilitate or hinder behavior change [3]; but unfortunately the small, two-wave dataset did not allow us to test for diffusion of behavior through the network.

In another study, we demonstrated the diffusion of behavior (*i.e.*, physical activity) through a newly developed friendship network. During the course of an afterschool intervention, new childhood friendships influenced routine levels of physical activity. The strongest influence on the amount of time a child spent in moderate-to-vigorous physical activity after school was the activity level of his or her immediate circle of friends (typically four to six children). Children consistently made adjustments to activity levels of 10% or more in order to emulate the activity levels of their friends and were more than six times more likely to adjust their activity level to that of their friends than to keep their activity level constant [13]. We demonstrated that children became either more active or more sedentary as they emulated the behaviors of those with whom they had formed friendship ties.

The GROW trial explores the concept that by bringing groups of parents together regularly, the potential exists to create new social networks that can influence health behaviors (physical activity, nutrition). Diffusion studies show that people who are well integrated into a community (have many ties) generally adopt behaviors earlier than those who are less integrated (have fewer ties) [10,25]. Thus, we are interested in intentionally facilitating the integration of study participants into their small groups during the first twelve weeks of the intervention. Our intention was to facilitate strong relationships between study participants, which we defined as being bi-directional and existing outside of structured class time, because we aim to test the extent to which a new social network can influence health behaviors over the course of a three-year randomized controlled trial. The Social Network Diagnostics Tool is designed to increase the likelihood that new social networks are formed during the intervention.

### A model for using social network data during group interventions to increase group cohesion

Because the GROW intervention was designed to build a new social network around study participants, we wanted to intentionally augment new social ties and increase cohesion during the intervention. To measure advice networks, discussion networks, and perceived cohesion, we administered a short one-page assessment to pilot intervention group participants during sessions four and twelve (the last session). We administered the baseline survey at session four for multiple reasons: based on our past experience with group-level interventions, we predicted that some new relationships between participants would be measureable by that time; we anticipated that we would need one week to collect data from participants who missed the group session and two weeks to process the social network data during the larger trial; and we wanted to maximize the number of sessions (out of atwelve-session curriculum) that would benefit from the network measurements.

**Figure 1:**
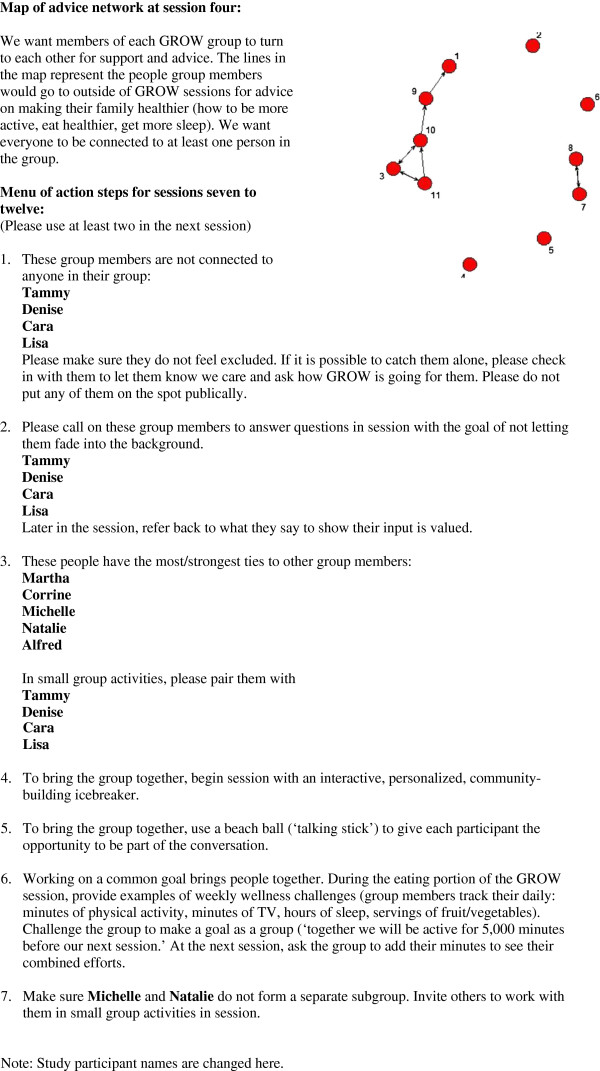
**Action plan for interventionist to increase small group cohesion.** Based on network diagnostic tool results.

Responses were analyzed and then discussed with the group leader. If a network was not forming or had structural signatures that seemed sub-optimal at session four, the group leader would be instructed to make adjustments to his/her teaching methods for sessions seven to twelve following a standardized protocol (Refer to Table 1). The group leader would receive a network map and specific data-driven recommendations on how to increase group connectivity (Refer to Figure 1). If the network was cohesive at session four, as defined by pre-defined thresholds per pre-determined network indicators (Refer to Table 1 ), then the group leader would be instructed not to alter his/her teaching methods. Table 1 outlines the metrics we used as diagnostics, why they were selected and what they mean, along with the corresponding 'menu of teaching methods’ to increase group cohesion in the new social network (our key mediator). We expected that at the beginning of the program, we would observe no or very few network linkages and no or a very low level of perceived cohesion among participating families who have no pre-existing relationship but come from the same zip code region.

We hypothesized (H_1_) that by week twelve, after weekly 90-minute group skills-building group sessions, we will observe a moderate increase in network structure and perceived cohesion among participants.

## Methods

### Data collection

During session four (week four) and session twelve (week twelve) online surveys were administered via REDCap (Research Electronic Data Capture) [26] at the community recreation center to the participants in attendance by trained study personnel. Study participants who were not in attendance on data collection days received a data collection telephone call within one week. Those who failed to complete the make-up survey by telephone had incomplete data but were not considered system-missing: while they did not provide network data (*i.e.*, nominate people with whom they had a relationship), they could have been nominated by other group participants. The study was approved by the IRB at Vanderbilt University (IRB#100591).

### Sample

Eleven (11) pilot study participants enrolled in a twelve-week intervention designed to teach healthy lifestyles in a group format. Eligibility criteria for study participation included: parent age ≥18 years; parent has at least one child three to five years of age with body mass index ≥50^th^ percentile and <95^th^ percentile; parent speaks English; parent has consistent phone access; parent resides in East Nashville as defined by zip code in proximity to the community recreation center; parent and child are healthy, without medical conditions necessitating limited physical activity as evaluated by a pre-screen; and parent and child are considered underserved, as indicated by self-report that that either the index parent or someone in the household participates in at least one of these government subsidized programs: TennCare, CoverKids, WIC, Food Stamps (SNAP), Free and Reduced Price School Lunch and Breakfast, Families First (TANF).

These eligibility criteria intentionally created a homogeneous group. Homophily, the tendency for people to associate with similar others [27], is commonly present in networks, and, homophilic relationships share common characteristics (beliefs, values, education, etc.) that make communication and relationship formation easier. Thus, homophily is a concept that underpins building group cohesion. In the pilot study of eleven participants, ten were female; nine were non-Hispanic African-American, one was White Hispanic, one was non-Hispanic biracial. Mean age was 30 (SD = 4, range 22 – 39). Eight participants completed the baseline survey. Of those, seven completed the follow-up network survey; the absent participants were called per protocol but did not answer their telephones to complete the survey. Two participants had a pre-existing relationship.

### Measures

#### Social network

A social network survey was developed to assess change in social relationships (specifically, advice networks and discussion networks) over the course of the study period by capturing the presence and absence of ties at mid-point and completion of the intervention. The items were: In your GROW group, who would you go to *outside of sessions* for advice on making your family healthier (like being more active, eating healthier, and getting more sleep)? (advice network); and in your GROW group, with whom do you discuss these issues (being more active, eating healthier, and getting more sleep) *outside of sessions*? (discussion network). We measured both advice and discussion networks in order to capture two components of interpersonal influence thought to be important, expertise, and trust [28]. We measured relationships outside of sessions in order to capture stronger personal ties, rather than the weaker formal associations artificially created in a structured classroom setting, where all participants are required to interact with each other.

To ease respondent burden and to reduce measurement error, participants were given a list of first and last names of group participants along with their photograph. The whole new social network was defined as all 11 pilot participants. Directional and bi-directional ties were included in analysis. While 'discussion’ suggests participation from both partners, both partners needed to nominate the other for a bi-directional relationship to be present.

#### Perceived cohesion

We also administered Bollen and Hoyle’s rigorously validated cohesion scale to determine if participants increased their perception of cohesion relative to the group [29,30]. Originally developed for the community level [29], this scale (min = 1, max = 7) has been adapted and validated for the small group context [30]. This six-item measure reflects two underlying dimensions of cohesion: sense of belonging and feelings of morale. Items are: I feel a sense of belonging to my GROW group; I feel that I am a member of my GROW group; I see myself as part of my GROW group; I am enthusiastic about GROW; I am happy to be in GROW; and GROW is one of the best health programs anywhere. Items one to three reflect Sense of Belonging; items four to six reflect Feelings of Morale. Responses are recorded on a 7-point Likert scale with the following anchors: strongly disagree, disagree, slightly disagree, neither agree nor disagree, slightly agree, agree, strongly agree.

#### Description of the intervention

Pilot participants were enrolled in a twelve-session lifestyle pilot intervention delivered weekly at a community center operated by the Department of Parks and Recreation. Transportation and childcare for siblings were provided to all study participants to overcome the most frequently cited barriers to study participation [31]. Participants received three small incentives after each wave of data collection, but did not receive remuneration for attending sessions. All sessions were conducted in English by the same group leader, who was trained to facilitate group discussion rather than lecture. All sessions involved a parent-only skills building component and a parent–child applied learning component to build healthy lifestyle skills (nutrition, physical activity). Integrated within our intervention was the intentional building of new social networks.

#### Description of the social network building component of the intervention

Using the data collected at session four and the pre-determined social network diagnostics and thresholds, we created an action report with concrete recommendations, tailored to the group dynamics at session four. This action report was then discussed with the group leader who was instructed to use its recommendations during sessions seven to twelve to increase group cohesion.

The action report included: a map of the advice network, where nodes were labeled with participant names; and a menu of action steps derived from the social network data collected at week four to guide teaching methods (*e.g.*, Connect Tammy with any of these four group members; Make sure Michelle and Natalie do not form a separate subgroup; etc.). Figure 1 shows the data-driven action report provided to the group leader in order to facilitate increased group cohesion during sessions seven to twelve. The group leader was instructed to implement at least two recommendations during each subsequent group session.

#### Analysis

We computed individual and network level metrics at two time points (session four and twelve): isolates, degree, reciprocity, sub-groups, density, centralization, transitivity, and cohesion. We expected change on all measures between measurement periods. This pretest-posttest design served to provide preliminary data on the feasibility of collecting data during an intervention and providing feedback to the group leader to increase group cohesion during the remaining intervention sessions. Psychometric analyses of the perceived cohesion scale and its change over time are also presented.

## Results

On average, participants attended seven of the twelve weekly sessions, with five participants attending eleven sessions and two failing to attend any sessions. At week four, eight of eleven participants provided network data. At week twelve, seven of eleven participants provided network data, resulting in two waves of data for seven of eleven participants. No data were imputed. If a participant did not participate in data collection, but was nominated by another participant as a discussion partner, a directional tie would have been included in the analysis. Of the four non-respondents, two did not attend any sessions, one attended one session, and 1 attended three sessions.

The action report resulting from the network data collected week four and provided to the group leader to use through week twelve is given as Figure 1. Those diagnostics that met our pre-specified thresholds were included in the menu of teaching recommendations provided in the action report. To provide a cogent illustration, we included only the action report based on the advice network here. The group leader received action items based on the discussion network as well.

**Figure 2:**
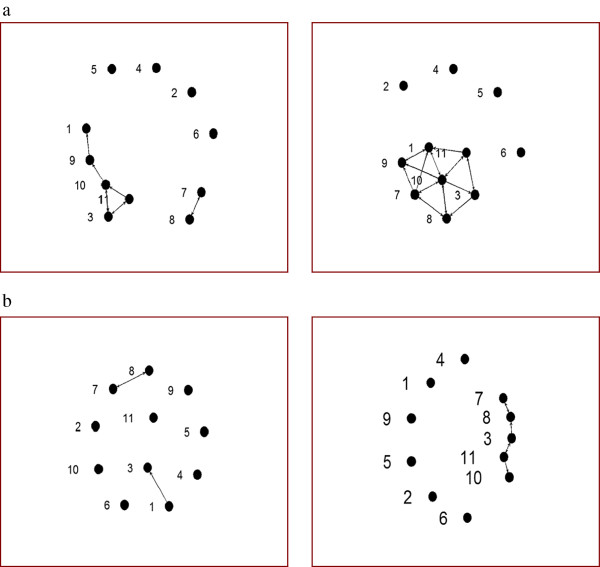
**Evolution of social networks during the intervention period. (a).** Advice networks at weeks four and twelve. **(b).** Discussion networks at weeks four and twelve. Density, the proportion of links in the network, increased from 0.082 to 0.182 in the advice network (t = 2.13, p < 0.05); and from 0.027 to 0.055 in the discussion network (t = 1.02, p > 0.05). This greater than two-fold increase represents a substantial shift in reported network partners over eight weeks of programmatic activity.

**Figure 3:**
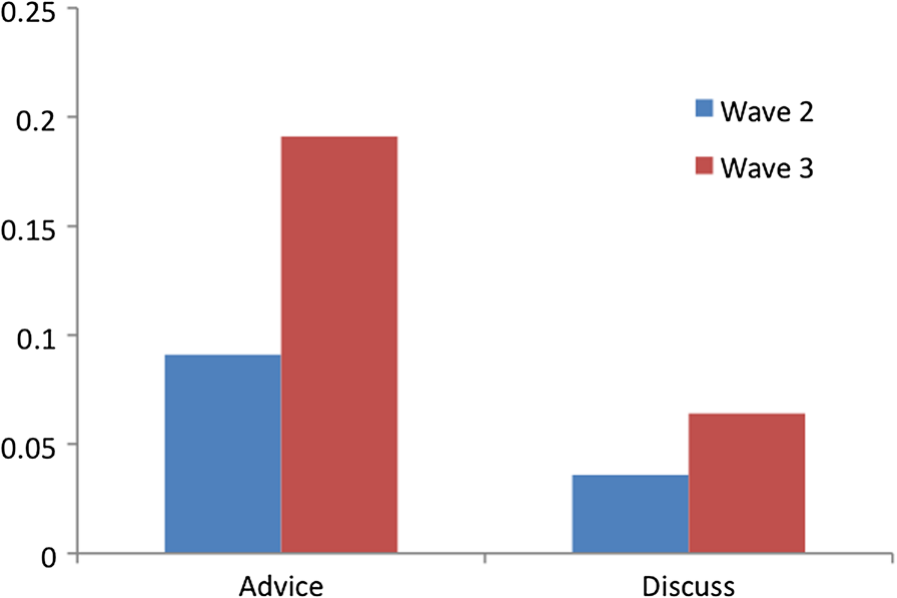
**Network density for advice and discussion networks at weeks four and twelve.** The density of the advice network and the density of the discussion network each increased from weeks four (wave two) to twelve (wave three). The number of reported advice partners and discussion partners increased but the density values are depressed by the four non-participants who did not provide nominations and were not nominated due to their non-participation.

As can be seen in Figures 2 and 3, the number of reported advice partners and discussion partners increased from weeks four to twelve. As the data in Table 2 show density, the proportion of links in the network, increased from 0.082 to 0.182 in the advice network; and from 0.027 to 0.055 in the discussion network. This two-fold increase represents a substantial shift in reported network partners over time. Using UCINET [32], we calculated a bootstrapped t-test that controls for the non-independence of the network data [33]. The advice network change was statistically significant (t-value = 2.13) but the discussion network was not (t-value = 1.02). The actual density values are depressed somewhat by the four non-participants who did not provide nominations and were not nominated due to their non-participation. Figure 2a and b show that there are four isolates (nodes 2, 4, 5, 6) in the advice network at week four who remain isolated at week twelve. Although the group leader was given specific recommendations to integrate these individuals into the group, their failure to attend sessions hampered such efforts. Figure 2a and b also show that the specific recommendation not to allow two group members to form a separate subgroup was effective. These individuals (nodes 7, 8) had a pre-existing relationship and had not formed ties to other group members by week four. At week twelve, however, they were integrated into the network. Other network indicators also changed over time and are shown in Table 2. These data provide proof of concept and suggest that using the Social Network Tool to empirically guide program activities of a childhood obesity intervention was feasible

**Table 2:** Network diagnostics from the GROW pilot data

**Week**	**Network**	**Size**	**Density**	**Reciprocity non-nulls**	**Centralization**	**Transitivity percent**	**Cohesion**
4	Advice	11	0.091	0.5	0.130	0	0.192
4	Discussion	11	0.036	0	0.100	0	0.108
12	Advice	11	0.191	0.538	0.240	0.018	0.336
12	Discussion	11	0.064	0.200	0.050	0	0.169

Multiple network metrics showed change in the predicted direction suggesting the group was moving toward increased connectivity. Absolute connectivity remains modest. These data serve as proof of concept.

Factor analysis of the perceived cohesion scale indicated that the six items loaded on one factor with an eigenvalue of 4.48 and a Cronbach’s alpha of 0.92. The mean score on the scale increased from 6.07 to 6.57 (F = 1.47; df = 1,12; p = 0.24). Although the change did not attain statistical significance, the values are in the predicted direction.

## Discussion

This paper presents a model and Social Network Diagnostic Tool for monitoring group dynamics during intervention implementation, and potentially improving program outcomes. Social network influences are a promising mediator of behavior change. Many behavioral interventions occur in a group setting, yet the underlying group structure and group processes are not documented in the scientific literature. The Social Network Diagnostic Tool is intended to be used to systematically monitor group programs during implementation and to empirically guide program activities with the intent of building new social networks.

GROW is an intervention that uses social network data to accelerate behavior change, thereby meeting the definition of a social network intervention [34]. Unlike any past study, however, in the ongoing GROW trial, social network data will be used as a diagnostic tool to aid intervention implementation. The pilot data presented here served as a proof of concept for the Social Network Diagnostic Tool. The GROW intervention is designed to construct a new social network around each intervention participant to aid behavior change and to sustain it over three years. By collecting network data mid-intervention and using the data to guide programmatic activities toward increased group cohesion, we aim to intentionally build and strengthen new relationships. It is through these new social ties that we hope to accelerate the diffusion of ideas and behaviors that support a healthy lifestyle. With the Social Network Diagnostic Tool, we can measure whether the intervention has resulted in a new social network, as intended. Thus, it serves as a treatment fidelity check. Ultimately, with multiple groups and multiple waves of network and behavioral data, it will be possible to assess if social network influences mediate program outcomes.

In this small pilot sample, density, the proportion of links in the network, doubled in both networks, with the increase in the advice network reaching statistical significance. This two-fold increase represents a substantial shift in reported network partners over time. While the Social Network Diagnostic Tool was developed for interventions that intentionally bring strangers together in a group format to form a new social network, it could just as easily be used in interventions that leverage existing social networks (*e.g.*, high school classrooms, work groups). While no currently validated threshold values exist, we set *a priori* thresholds based on our prior experience and expert guidance as a starting place, and in order to standardize our protocol and remove subjectivity from data interpretation. For example, the relationship between diffusion of ideas or behavior and density is likely to be curvilinear, with some density necessary for diffusion to occur, more density capable of accelerating diffusion, and too much density being redundant and inefficient [3]. In dense networks, people are more interconnected and health information and behaviors can spread more rapidly and to more people [20]. In sparse networks, there are few pathways for information and behaviors to flow. Yet networks that are too dense may hamper group performance [35]. Integrating several decades of research, Valente proposes that optimal levels of density likely lie between 0.15 and 0.50 [3]. Thus, we use these thresholds in the Social Network Diagnostic tool, recognizing that the optimal level of density for a group probably varies by group characteristics and the kind of behavior change desired. Researchers who use the Social Network Diagnostic Tool in interventions that rely on existing networks may choose to adjust these thresholds upward to be more sensitive to the structural characteristics within their particular networks. Future research is needed on the distribution of these metrics during interventions that can better inform threshold values.

The manner in which diagnostics estimates trigger particular teaching recommendations is quite flexible. To illustrate, observed low degree might prompt recommendations that are data-specific (*e.g.*, 'pair Betty with Mary in the next small group activity’), or general (*e.g.*, 'begin the next session with an ice-breaker activity to get all participants involved’). We feel, however, that it is important to specify the thresholds and branching logic (if measure x is low, then recommend a, b, and c) in advance, so that the tool can be consistently implemented across multiple groups over a long trial period.

It has been demonstrated that among discussion networks, problem-focused discussion networks are the most influential in the achievement of specific outcomes. Social networks of individuals with whom one discusses 'important matters’ may not be predictive of health outcomes, whereas social networks of individuals with whom one discusses 'health matters’ are indeed predictive of a wide range of health and health services-related outcomes [36]. Perry and Pescosolido compared multiple egocentric social networks of people experiencing an acute health crisis and evaluated their relative influence on health outcomes: those ties with whom important matters are discussed, but not health; those ties with whom health is discussed, but not important matters; and those ties whom both important and health matters are discussed [36]. They found that the discussion networks focused on health matters explained a wide range of health and health services-related outcomes, whereas the discussion networks focused on important matters did not. Thus, other researchers who use the Social Network Diagnostic Tool would be advised to modify the name generator questions to ask respondents to nominate those individuals with whom they discuss the outcome of interest. To illustrate, members of a smoking cessation support group would be asked about individuals with whom they discuss smoking/tobacco products if the outcome of interest is smoking cessation.

Centralization increased in our advice network and decreased in our discussion network. Centralization is a measure of the extent to which pathways in the network are connected to a small number of nodes. In the advice network at week four, one node has a relatively higher number of incoming and outgoing ties; by week twelve this node has become even more central to the network with direct ties to all connected group members. Centralization increased in this advice network primarily because isolates at week four became integrated into the network by week twelve. In contrast, in the discussion network at week four, one directional tie existed. By week twelve, new ties were evenly distributed among several nodes forming a decentralized chain.

The perceived cohesion scale (PCS) used in the present study was validated for small groups and thus appropriate for this setting. Although the PCS was validated for small groups (with 70 groups of four to five members per group, n = 330 [30]), the sample size used in this proof of concept was too small to perform a factor analysis on, and may therefore have resulted in a one-factor solution. The change on the PCS was non-significant, likely due to the small sample size and observed ceiling effect. Our data collectors felt quite strongly that the high scores and limited variability in the PCS at both baseline and follow-up was a result of respondents providing social desirable responses to study team members. It is due to the small sample size that we did not correlate network measures and self-reported cohesion. To the best of our knowledge, only one paper examines the relationship between social network metrics and perceived group cohesion [37]. That study showed that individual sociometric choices and group-level sociometric cohesiveness had moderate, significant positive correlations to self-reported cohesion. Soldiers who were more often nominated by their squad members felt there was more cohesion in their squad. Squads in which members made more in-group nominations were also more cohesive.

### Limitations

There are many different mechanisms that might explain the social influences of social networks: diffusion of information, conformity to group norms, social comparison, social learning, imitation, coercion, competition, etc. [38,39]. At this time, this tool is not sensitive to measuring these processes or their relative influence in this or other settings. Once we better understand the underlying behavior change mechanisms, future extensions of this tool can leverage these particular social influences/mechanisms and potentially strengthen the intervention effects.

Attrition is the greatest challenge to the group leader fully implementing the recommended action items. When participants do not attend sessions, it can be impossible to implement recommendations that relate to those individuals (*i.e.*, the group leader can’t pair Betty with Mary if Betty fails to show up). In our pilot data, several isolates at week four remain isolates at week twelve. The action report included strategies to integrate these individuals into the small group, but because they did not attend sessions, the group leader could not implement the recommended strategies. Indeed, it is likely that the participants with the lowest session attendance will also be less connected to other group members and be more likely to be included in specific teaching recommendations. We tried to temper this vicious cycle by providing multiple recommendations and requesting the group leader attempt to implement at least two of them in each session. The observed change in network measures over time supports this approach.

## Conclusion

Given that many behavioral interventions occur in group settings, intentionally building new social networks could be promising to augment desired outcomes. We present a Social Network Diagnostics Tool and proof of concept: using this tool to empirically guide program activities of a childhood obesity intervention resulted in increased group cohesion. This tool represents a new way to capture data during treatment to inform treatment, and has the potential to lead to a new model for behavioral group setting programs.

## Abbreviations

SNA: Social network analysis; GROW: Growing right onto wellness.

## Competing interests

The authors declare that they have no competing interests.

## Authors’ contributions

All authors made substantial contributions to study conception and design, data collection, data management, and interpretation of data. TV wrote the network analysis program and analyzed the data. SG and TV drafted the article and revised it critically for important intellectual content. SB revised the article critically for important intellectual content and supervised the research group. All authors read and approved the final manuscript.
